# HRSBallast: A high-resolution dataset featuring scanned angular, semi-angular and rounded railway ballast

**DOI:** 10.1016/j.dib.2020.106471

**Published:** 2020-11-01

**Authors:** André Broekman, Jacobus Oostewald Van Niekerk, Petrus Johannes Gräbe

**Affiliations:** Department of Civil Engineering, University of Pretoria, South Africa - University of Pretoria, Lynnwood Road, Hatfield, Pretoria 0002, South Africa

**Keywords:** Railway ballast, Digitised ballast, VSLAM, Discrete element modelling, Blender

## Abstract

A high-resolution dataset of digitally scanned railway ballast (HRSBallast) is presented, consisting of 108 individual, digitised samples. The ballast samples were sourced from both a railway test track section located on a heavy haul coal export line in South Africa and a local quarry. The sampled ballast from the field installation represent angular, semi-angular and rounded geometric features depending on the sampled location. The fresh ballast sourced from the quarry was iteratively tested using a hydraulic actuator in a small-scale box test which forms part of a separate research project. The ballast samples were scanned before and after every test to ascertain the changes in geometry, in addition to the loss of material due to attrition. The field and laboratory samples were digitised using a high-resolution, commercial (Visual Simultaneous Localization and Mapping) VSLAM-based scanner with a 40-micrometre accuracy. Samples which were fractured by the hydraulic actuator are also included in the dataset. HRSBallast serves as a reference dataset for granular media (GM) simulations utilizing DEM (discrete element method), degradation or wear modelling, digital assets for the creation of synthetic datasets for deep learning applications, embedded railway instrumentation and video games requiring high-resolution geometry.

## Specifications Table

**Table:** 

Subject	Railway Engineering
Specific subject area	High-resolution, digital models of railway ballast comprising a range of geometry characteristics
Type of data	3D model geometry Image Table
How data were acquired	A commercially available EinScan Pro HD (Multifunctional Handheld 3D Scanner) manufactured by Shining3D® was used to scan all the samples described. The software (provided by Shining3D® together with the scanner) was used for calibration of the scanner, to perform the scans (using the included turntable), meshing and decimation of the final point cloud, and exporting the digital model as a STL (stereolithography) file.
Data format	Raw
Parameters for data collection	The first set of samples comprise angular, semi-angular and rounded ballast, sampled from an existing field installation subject to heavy haul loading conditions. The second set of data comprises fresh ballast that was rounded using a standard concrete mixer for a set period of time, prior to installation in the laboratory box test and subjected to cyclic loading.
Description of data collection	To create a digital model of the ballast samples collected from either the field installation of sourced from a local quarry, the samples were first washed and dried to remove any fouled material from the surface, followed by numbering with a silver, acrylic-based marker before being scanned. Every ballast sample was photographed, including before and after every box test (where applicable) that investigated the material loss as part of a secondary study. The samples were weighed using a calibrated laboratory scale with a resolution of 10 mg.
Data source location	Institution: Department of Civil Engineering, University of Pretoria City: Pretoria (fresh ballast samples) and Bloubank (field ballast samples) Country: South Africa
Data accessibility	Repository name: Mendeley Data Data identification number: 10.17632/5txdfwdypb.2

## Value of the Data

• The dataset (HRSBallast) provides high-resolution, digital models of railway ballast obtained from representative and in-service environments which are both difficult to recreate and not readily available in the public domain.
• HRSBallast can be used as a reference dataset for DEM, degradation and wear modelling, the creation of synthetic datasets required for deep learning applications [1] and embedded railway instrumentation [2].
• Using realistic railway ballast geometry accelerates the development of numerical simulations - specifically for DEM - improving the accuracy of the simulation results. No datasets are readily available with practitioners relying on in-house scanning solutions to generate their own samples [3].

## 1 Data Description

**Fig. 1:**
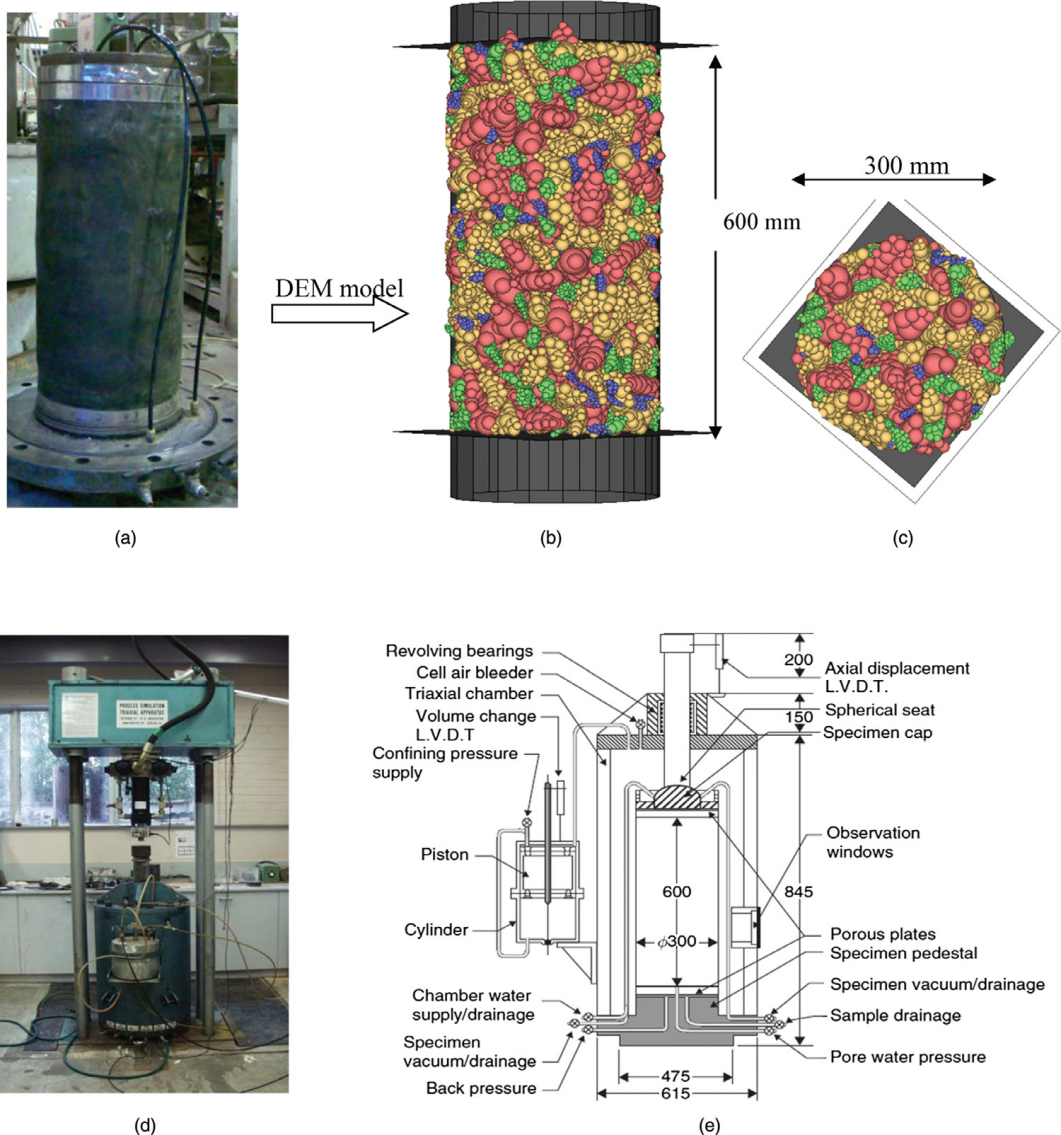
Example of tri-axial testing of a ballast sample (a) in the laboratory, a (b) and (c) planar view of the equivalent DEM model and a (d) photograph and (e) schematic of the full-scale test configuration [11].

**Fig. 2:**
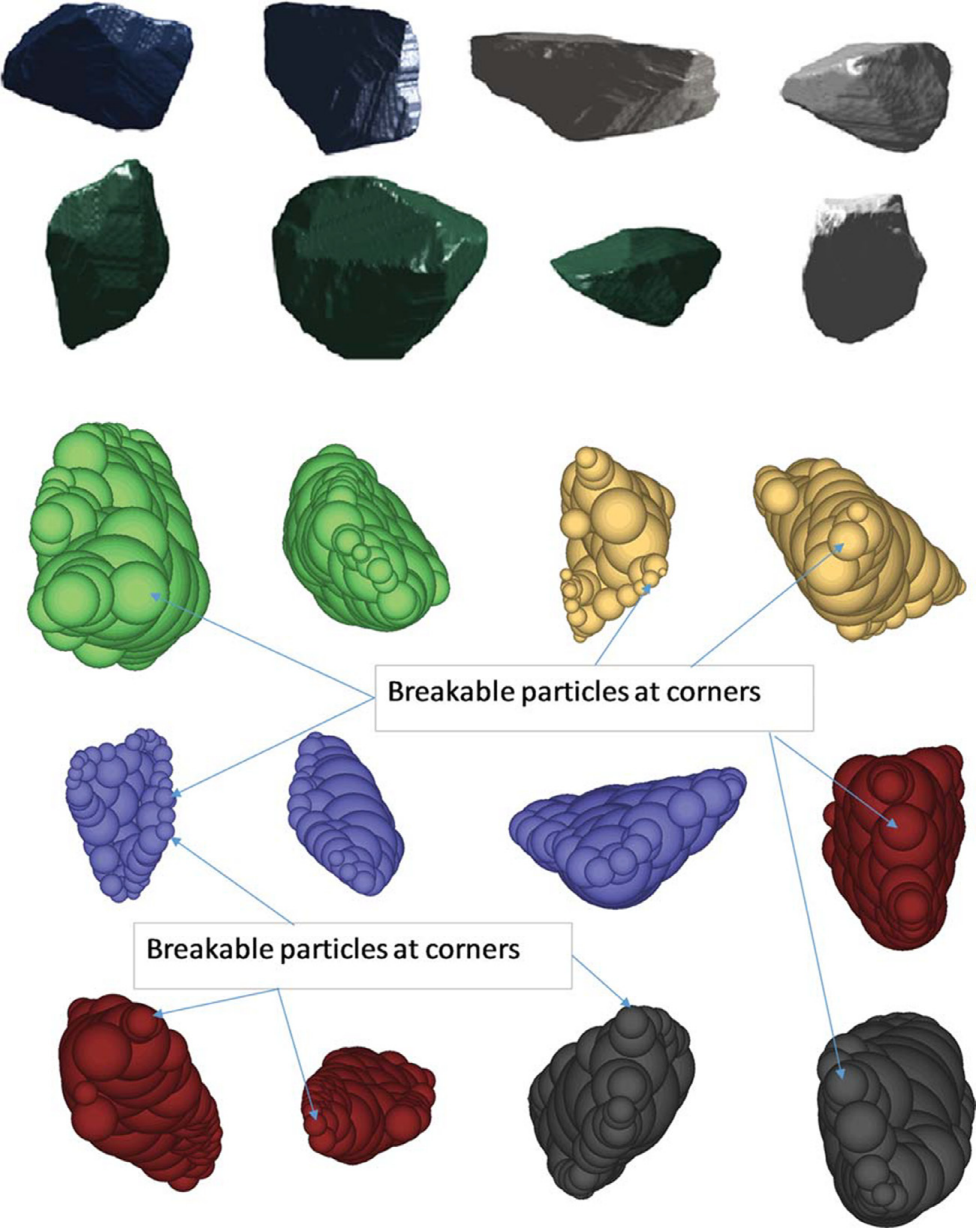
Example of digitized ballast samples (top) and simulated ballast particle shapes used in DEM (bottom) [10].

Railway infrastructure is designed for the economical and safe transportation of passengers and freight. The rail structure with its defined vertical and horizontal alignment, in combination with the various track components, provides the required performance subject to a range of environmental and loading conditions. The response of the granular material supporting the superstructure results from a complex interaction of principal stress rotation [4], plastic settlement, fouled material and dynamic loading conditions. Statistical descriptors using applied information theory [5] and entropy [6,7] provide the theoretical framework, relating the continuum concepts of granular media to the mesoscale behavior of the discrete components. The mechanical behavior of the ballast is primarily investigated using a combination of experimental and numerical modelling [8]. New sensing technologies such as MEMS-based (Micro-Electro-Mechanical-Systems) accelerometers provide new avenues to investigate the mesoscale behavior of discrete media [2]. The incorporation of highly parallelized GPUs (graphical processing units) by DEM software [9] has seen the increased adoption of numerical investigation of granular media and assemblies [10,11] which cannot be described by closed form solutions (Fig. 1). In contrast to the readily available physical mechanical properties of railway ballast [3], representative digital models of the particles are not available. Instead researchers typically rely on their own methods and instrumentation [10] to generate a statistically significant number of suitably sized [12] samples of railway ballast (Fig. 2).

For the proposed dataset (available from the corresponding online data repository [13]), the railway ballast samples (108 in total) were collected from two locations:

• An instrumented section of railway track on the Coal export line near Bloubank, KwaZulu-Natal in South Africa [14]. This section of railway track forms part of an in-service line (26 tonnes per axle) used to transport coal from the Mpumalanga coal fields to the port in Richards Bay. These samples (45 in total) are denoted with a “F”-prefix, and
• Fresh ballast (virgin ballast material which has not been used in service) sourced from a nearby quarry in Pretoria, South Africa. These samples were subjected to accelerated testing in a laboratory. These samples (63 in total) are denoted with a “M”-prefix.

### 1.1 Field ballast samples

**Fig. 3:**
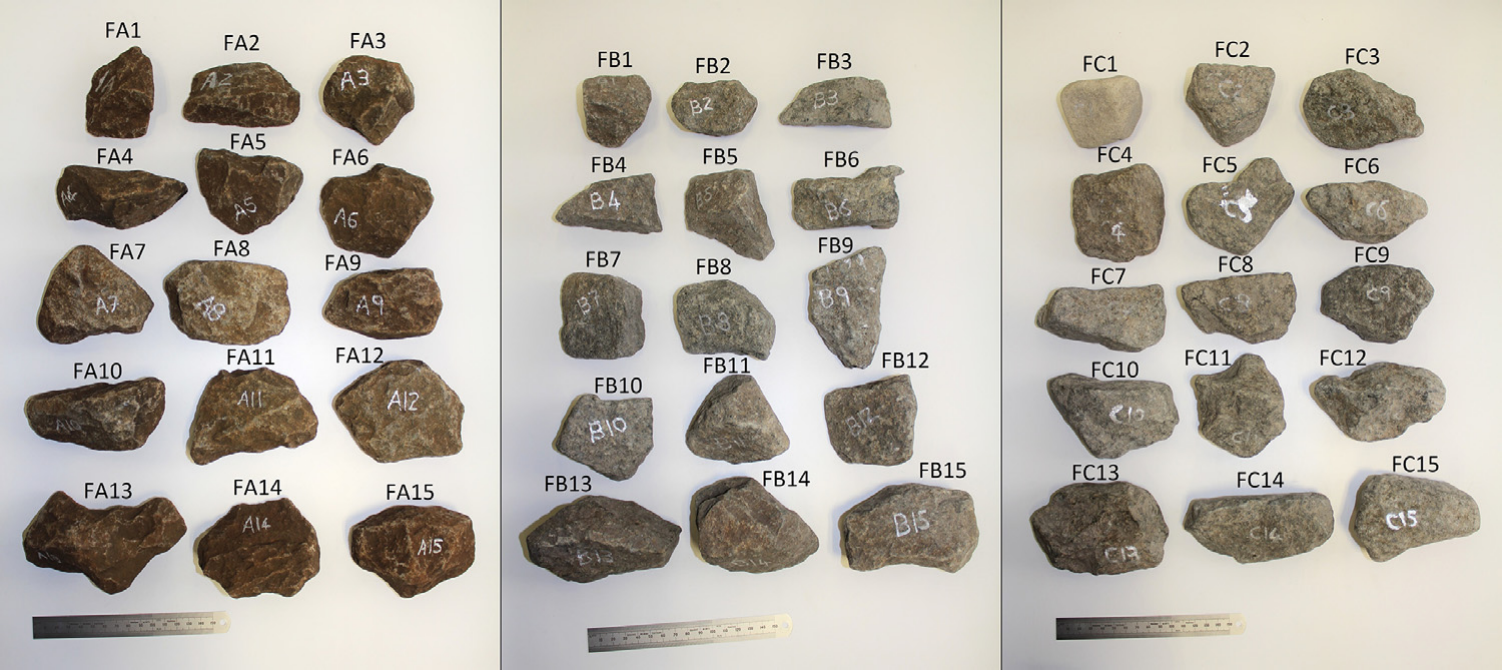
Ballast samples collected from the instrumented track on the Coal export line near Bloubank, South Africa

The 45 ballast samples collected from the instrumented track are sub-divided into three classes consisting of 15 samples each (Fig. 3). These are denoted as FA1 to FA15, FB1 to FB15 and FC1 to FC15 for the samples exhibiting angular, semi-angular and round geometrical features, respectively. Every sample's corresponding digital model (STL file format) is named according to the sample identifier, i.e. sample FA1’s corresponding filename is *FA1.stl*.

### 1.2 Fresh ballast samples

**Fig. 4:**
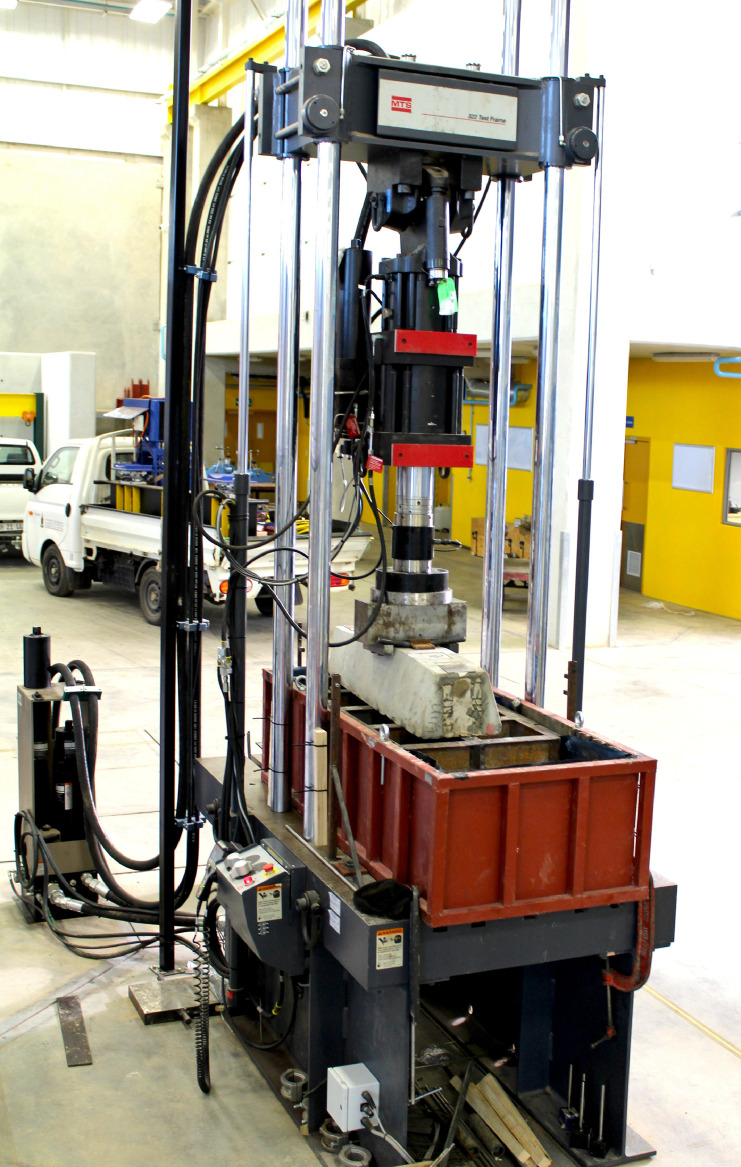
Hydraulic actuator used for accelerated testing of the ballast samples.

**Fig. 5:**
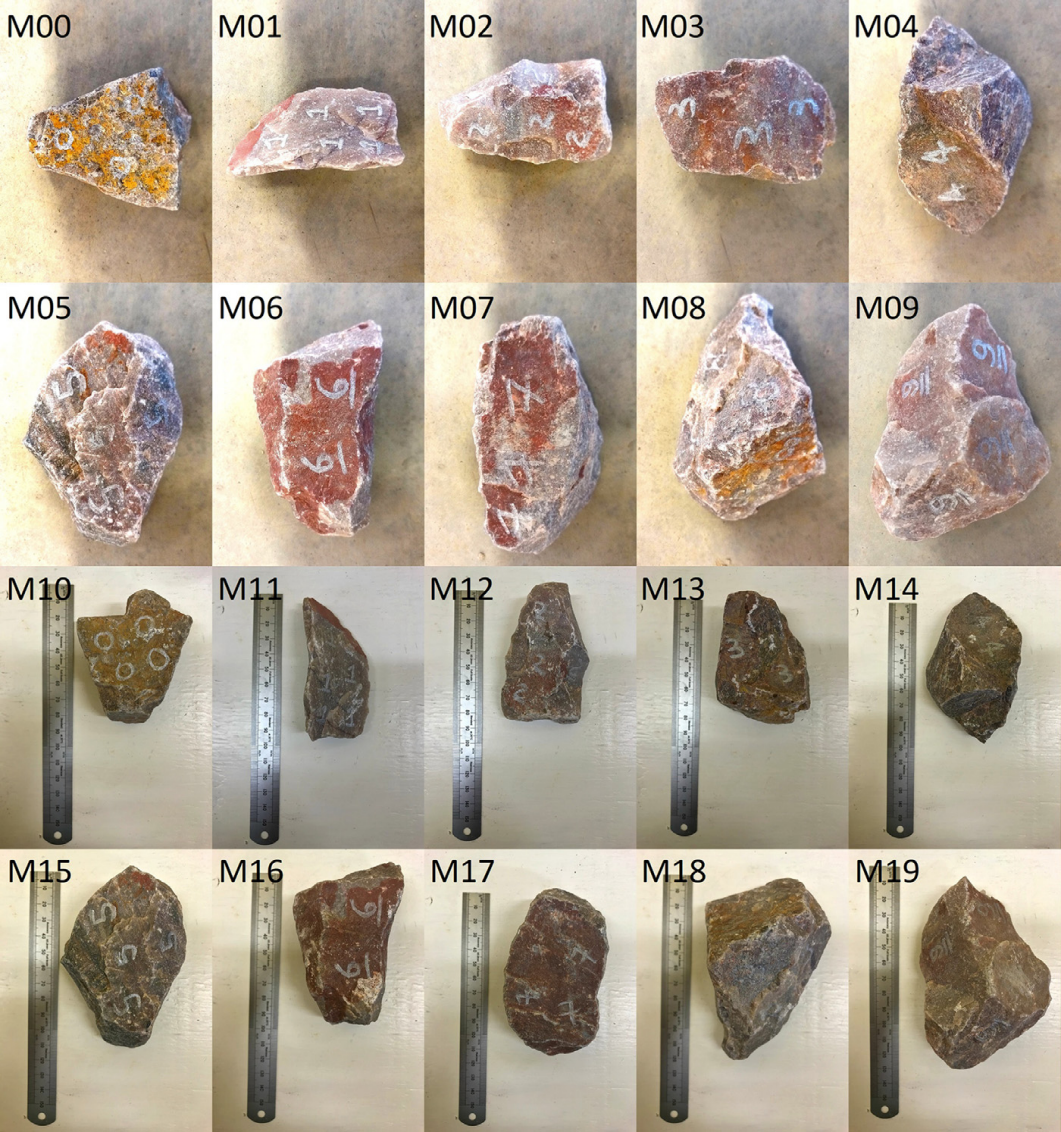
Collection of ballast samples with no prior treatment, before (M00-M09) and after (M10-M19) accelerated testing.
• Fresh ballast placed in a concrete mixer for 15 min (M0A-M0J). The samples were installed in the small-scale box test and subjected to 100,000 load cycles by the hydraulic actuator. The samples were scanned again after concluding the cyclic loading (M1A-M1J) (Fig. 6).

The 60 ballast samples obtained from the quarry are subdivided into three categories:

• Fresh ballast (M00-M09) installed in the small-scale box test and subjected to 100,000 load cycles by the hydraulic actuator (Fig. 4). The samples were scanned again after the concluding the cyclic loading (M10-M19) (Fig. 5).

**Fig. 6:**
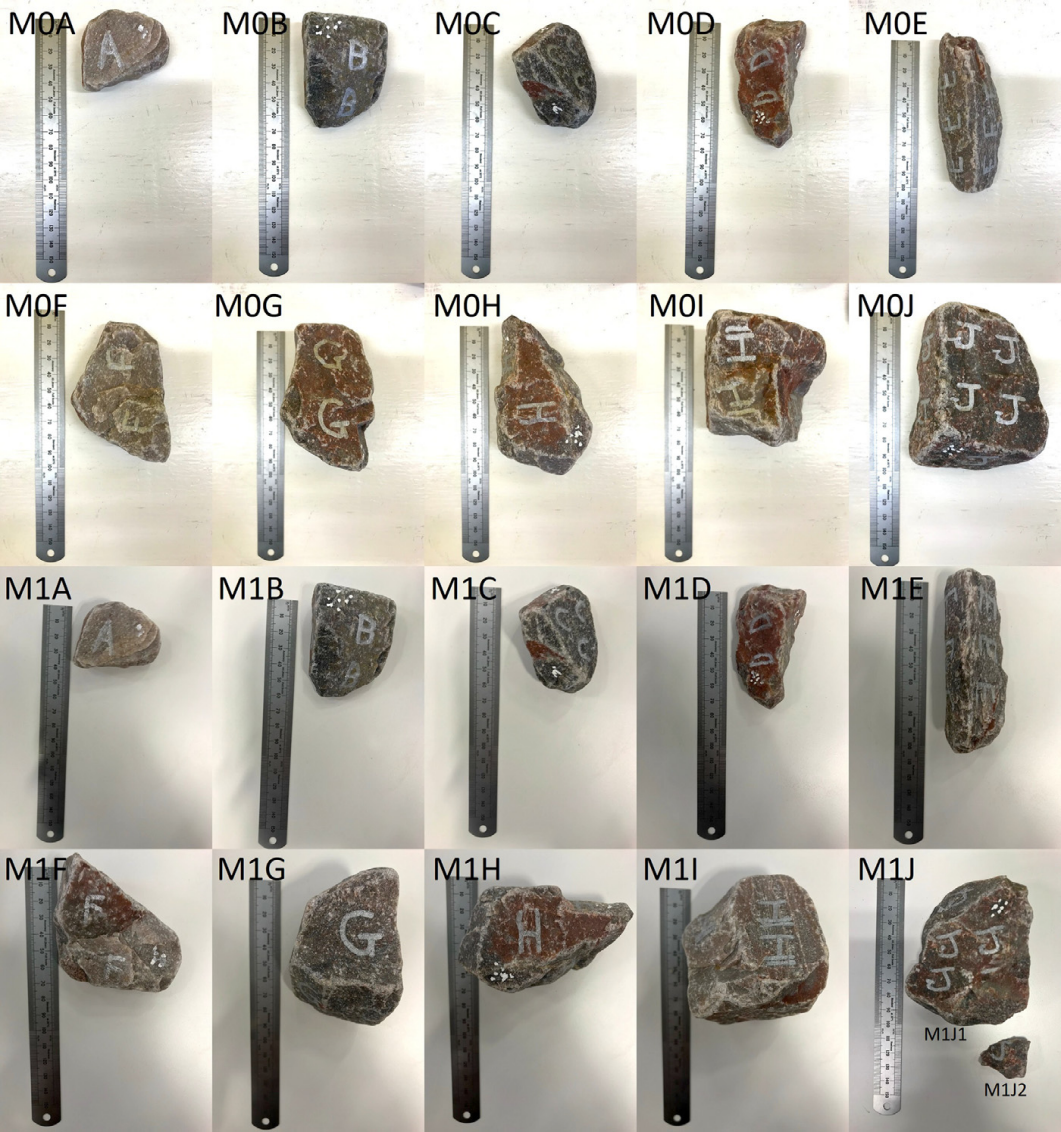
Collection of ballast samples with a 15 min pre-treatment, before (M0A-M0J) and after (M1A-M1J) accelerated testing.
• Fresh ballast placed in a concrete mixer for 30 min (M0K-M0T). The samples were installed in the small-scale box test and subjected to 100,000 load cycles by the hydraulic actuator. The samples were scanned again after concluding the cyclic loading (M1K-M1T) (Fig. 6).

The two collections of samples which were pre-treated in the concrete mixer (M0A-M0J, M0K-M0K) produced an additional 3 ballast samples (M1J, M1J2, M1T) originating from ballast fracturing during the cyclic loading. The fragments which were large enough to be successfully scanned are included as separate fragments (M1J: M1J1, M1J2; M1O: M1O4, M1O6; M1T: M1T1, M1T2). The addition of the sample fragments resulted in a total of 63 scans.

### 1.3 HighRes and LowRes

The original, high-resolution sample scans are available as a single archive (*HighRes.zip*) [13]. On average, a single model is comprised of 1.239 million vertices, measuring 118 Mb in size. In the interest of reducing the file size, the colour information associated with the point cloud was discarded and not included in the model file. The high-resolution models were simplified using Blender's decimation modifiers with a factor of 0.01 (1%) to produce a low-resolution version of the sample, aiding in the usability and application of the data for applications not requiring a substantial level of detail. This decimated collection of scan data is available as a single archive (*LowRes.zip*) archive [13].

### 1.4 Ballast_properties

The *Ballast_properties.xlsx* spreadsheet (with an equivalent *Ballast_properties.csv* file provided) summarises the following information:

• sample identification number (*Sample ID*);
• total number of vertices (*Vertex count*);
• total number of faces (*Face count*);
• *Volume* and *Surface area* as determined using Blender of the scanned sample;
• measured *Mass* using a calibrated laboratory scale;
• approximated *Density* by dividing the Mass by the Volume, and
• file *Size* of the high-resolution scan.

### 1.5 SampleMedia

**Fig. 7:**
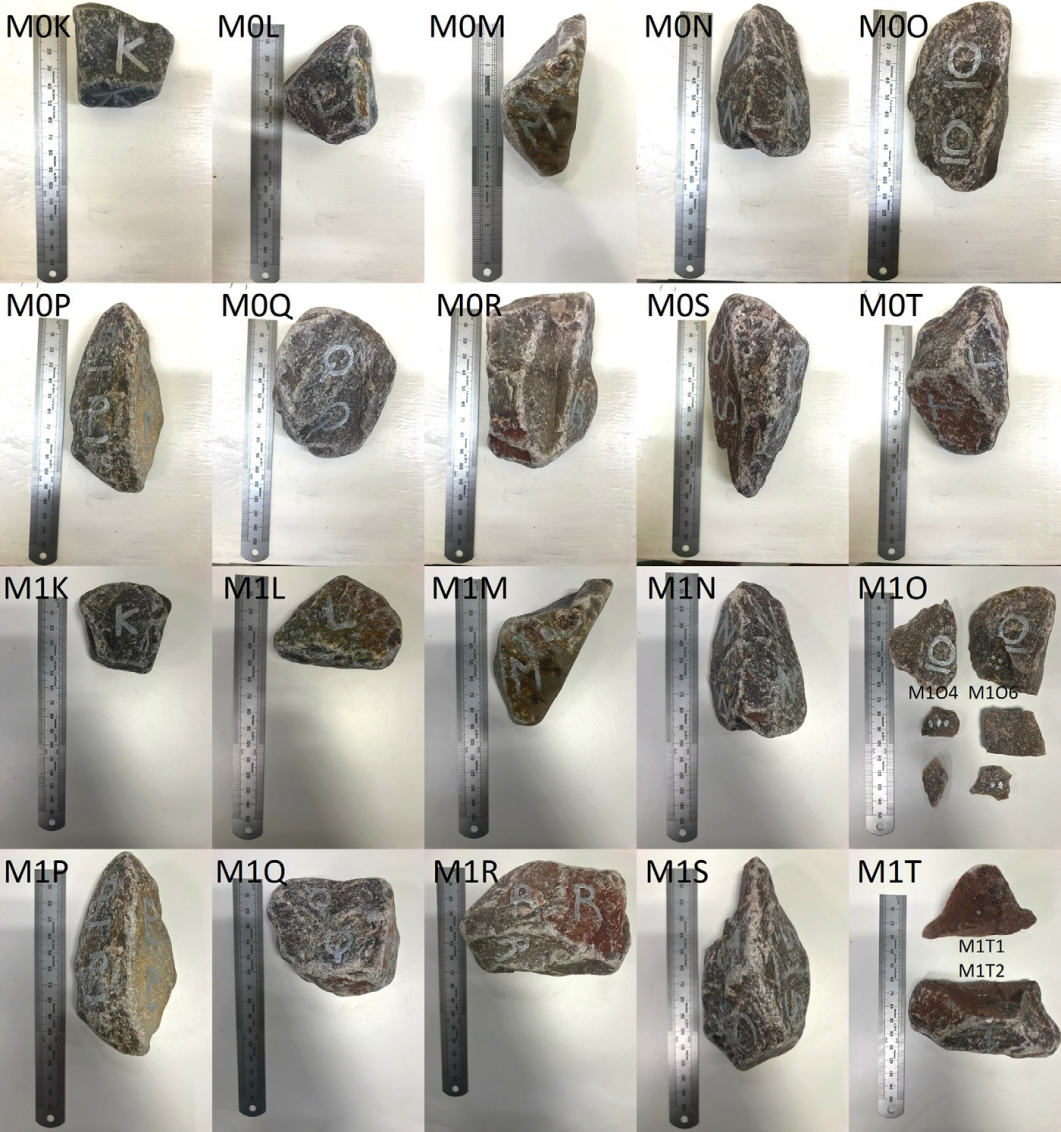
Collection of ballast samples with a 30 min pre-treatment, before (M0K-M0T) and after (M1K-M1T) accelerated testing.

High-resolution photographs (JPG format) of every ballast sample are provided in the data repository [13]. Every photograph is named according to the corresponding scan file of a particular sample, e.g. sample FA1 corresponds to the scan and photograph file *FA1.stl* and *FA1.jpg* respectively. A collage of each sample collection is provided in the folder which corresponds to Fig. 3 through Fig. 7 in the article.

## 2 Experimental Design, Materials and Methods

**Fig. 8:**
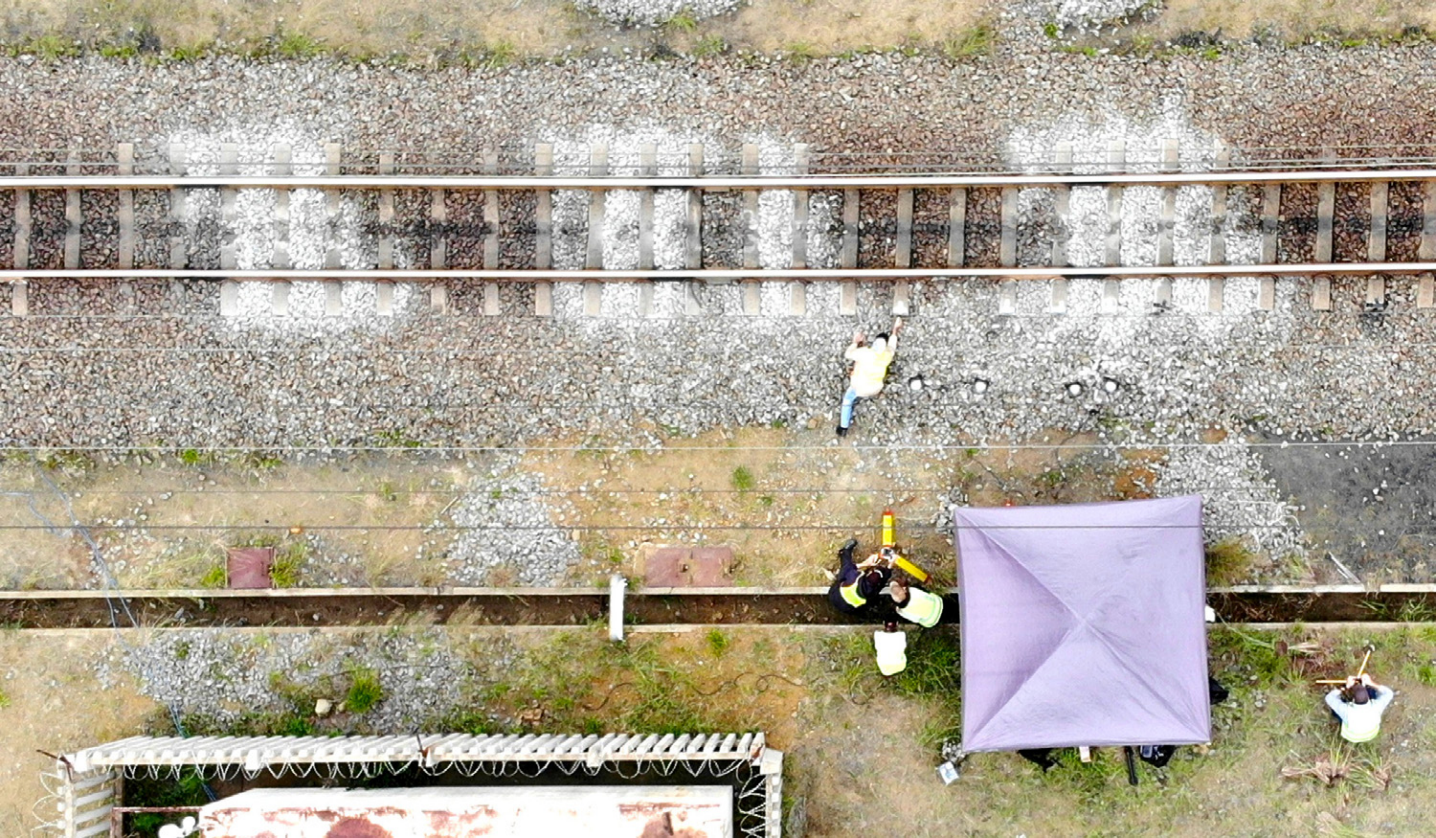
Overhead view of the instrumented section of rail track located near Bloubank.

**Fig. 9:**
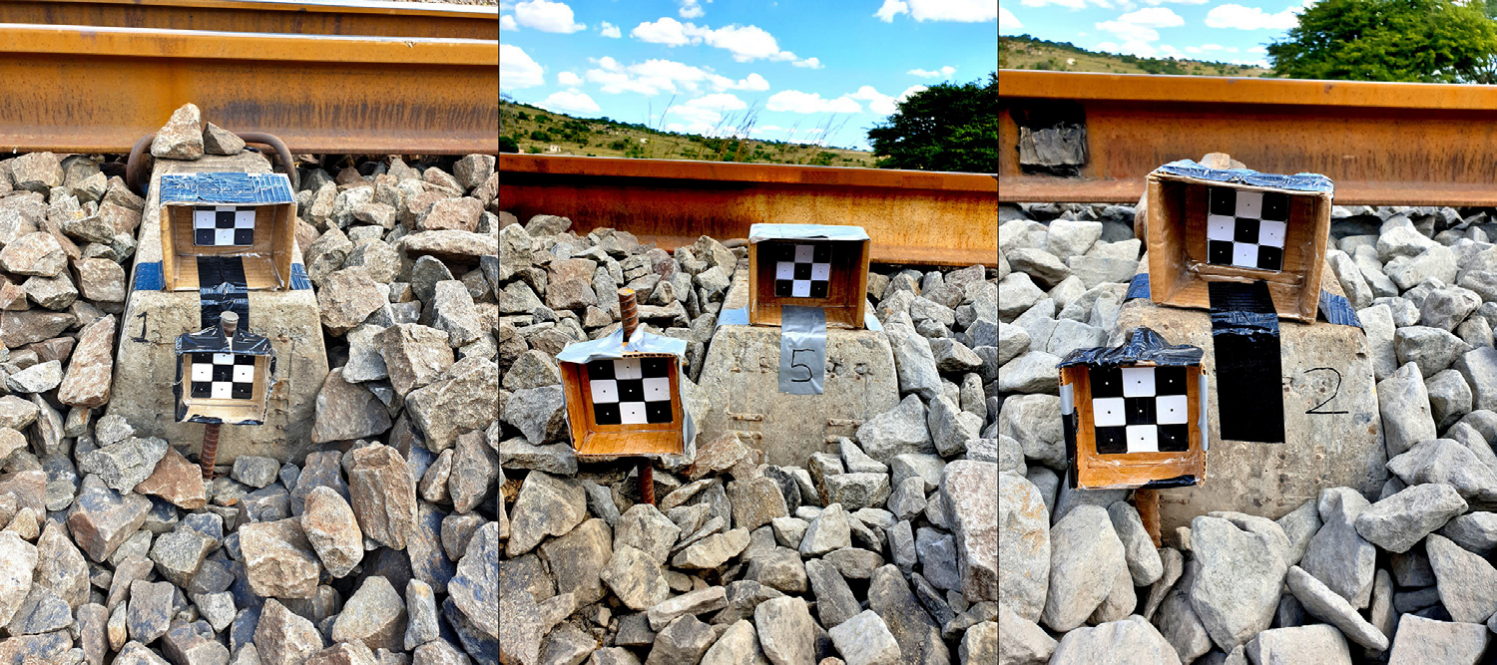
In-situ ballast sample representing angular (left), semi-angular (middle) and round (right) geometric characteristics.

The in-service ballast samples were obtained from the instrumented section of rail track located on the heavy haul Coal export line near Bloubank in the KwaZulu-Natal province of South Africa (Fig. 8). This natural distribution of geometric characteristics was encountered over a relatively short section of track, owing to the variability of the track structure support (Fig. 9). Note the association between the discolouration of the ballast (white, powdery surface) and the rounded geometry. The ballast samples – 15 samples from each of the three locations – were randomly sampled from the ballast shoulder of the railway track. The remote video monitoring (RVM) targets which are visible in the photographs, forms part of a parallel study relating the dynamic track performance to the ballast properties.

The fresh ballast samples sourced from the quarry were rounded for a predetermined amount of time using a concrete mixer. The inter-particle attrition serves as an efficient method to grind down the edges of the ballast. All the samples presented in this dataset conform to the specifications as required for South African heavy haul railway operating condition [12]. The ballast samples were thoroughly sieved, washed and dried to remove any soil, fouled material and organic matter adhered to the surface. A silver coloured, acrylic-based marker (*SOLO Goya TRITON acrylic paint marker 1.4 mm silver*) was used to clearly mark every sample with a unique (number of letter) designation across multiple locations on the surface. The acrylic paint is resistant to abrasion and does not compromise the scanning performance. Furthermore, the reflective markings of the paint assist the scanner in accurately aligning the discrete scans. The samples were weighed using a calibrated laboratory scale with a resolution of 10 mg. The mass of every sample, combined with the volume from the scanning process, yields an accurate estimation of the material density.

**Fig. 10:**
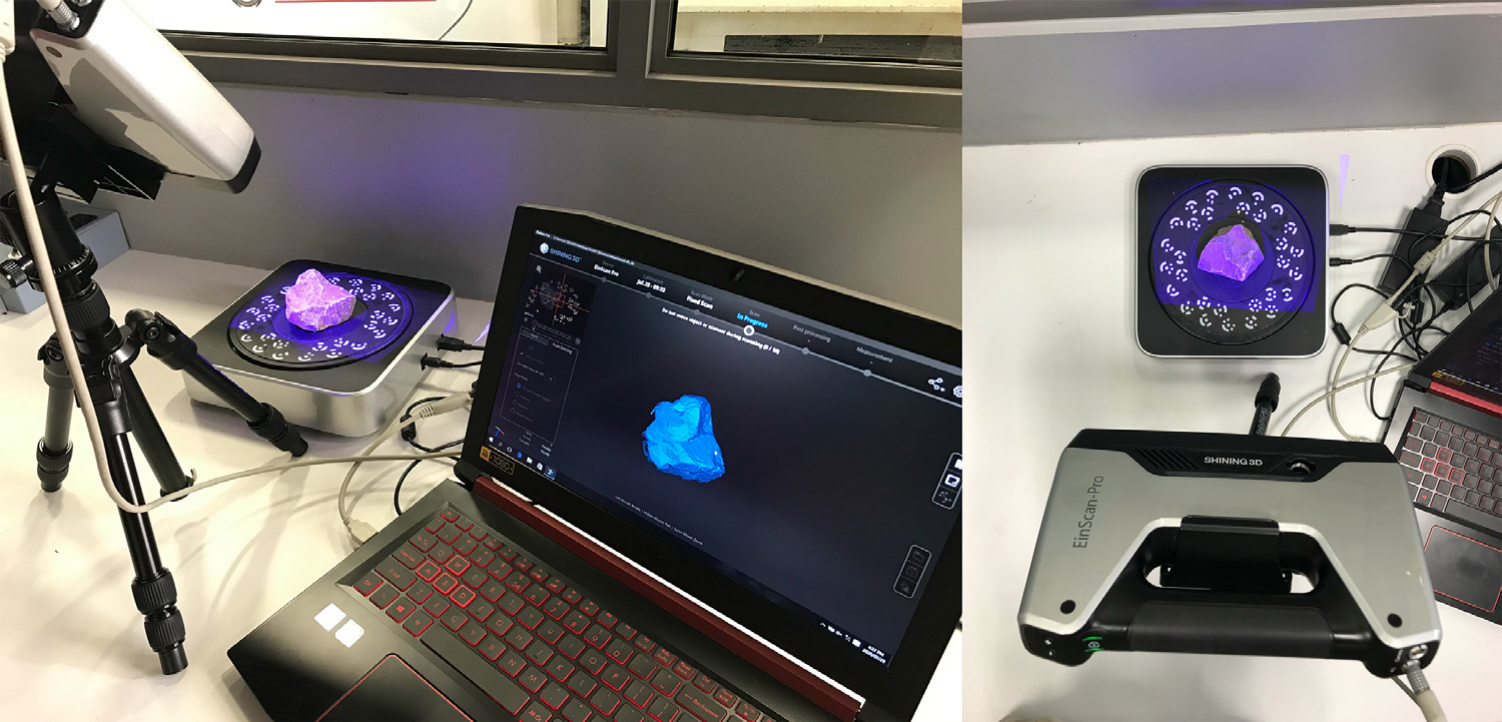
EinScan Pro scanner alongside the coded turntable and laptop computer.

**Fig. 11:**
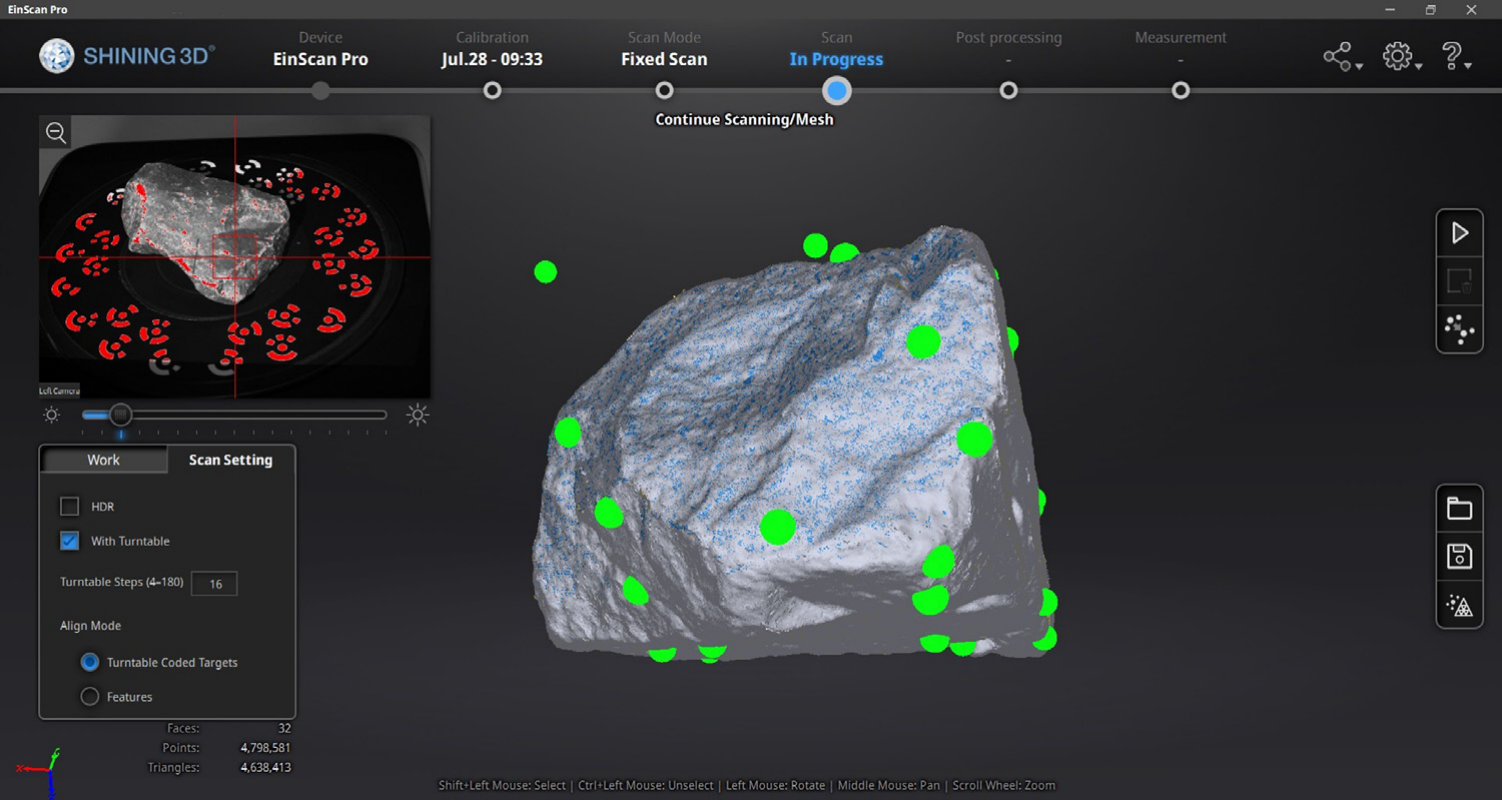
Screenshot of the software processing the point clouds during the scanning process.

A commercially available VSLAM-based EinScan Pro HD (Multifunctional Handheld 3D Scanner) [15] manufactured by Shining3D® was used to scan (digitize) all the samples described (Fig. 10). The fixed-scan method provides an accuracy of 40 micrometres and a point resolution of 240 micrometres. These specifications exceed that reported in recent literature [3] which report accuracies ranging between 100 and 220 micrometres depending on the scanner's orientation axis, emphasizing the value of the HRSBallast dataset. The scanner was calibrated prior to scanning the samples using the guided procedure provided by the manufacturer together with the certified calibration plate. The digitisation procedure consisted of either two or three orientations (depending on the geometry) of the sample, ensuring that the complete surface area of the sample is digitised. For every orientation of the ballast sample, the turntable rotates a total of 360 degrees in discrete steps (16 steps per full rotation were used for this dataset), scanning the visible surface at every step. A demonstrative video is included in the data repository to illustrate this process [13]. The software continuously combines the individual, smaller scans into a single, larger point cloud. After the turntable has completed a full rotation, the sample is reorientated until the entire surface area of the sample has been covered by the scanner. After scanning, the point clouds associated with every orientation is automatically reorientated and fused by the software to form a single, aggregated point cloud (Fig. 11). The ring coded targets etched onto the turntable ensures accurate reconstructions of the ballast. Prior to meshing (creation of an enclosed, manifold surface from the point cloud), the aggregated scan was visually inspected to ensure the seams were aligned correctly with all the edges sufficiently defined. The aggregated point cloud was meshed using the *medium quality* preset, prior to the decimation step. The generated mesh of every ballast sample was decimated (the removal of randomly selected faces from the model), limiting the number of faces to between two and three million for some measure consistency among the various ballast sample sizes. The final mesh was exported as a single STL file and stored on the computer. No scaling factors were applied during the last step of the export procedure, ensuring that the true dimensional scale of the model is retained.

**Fig. 12:**
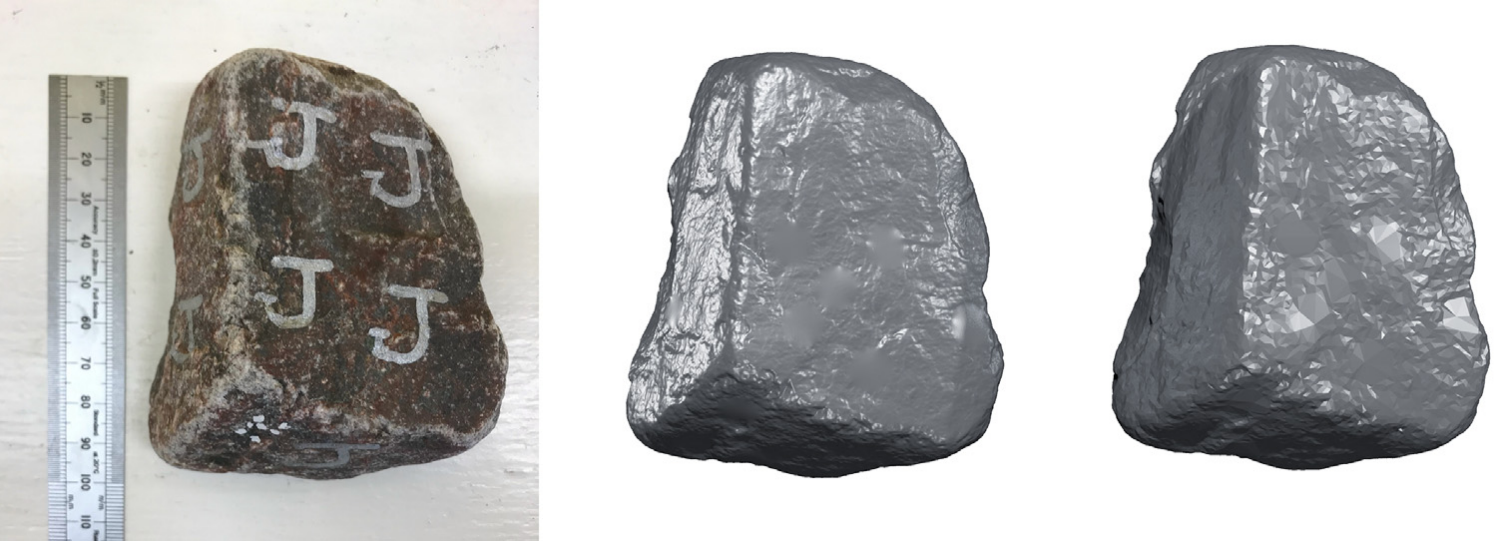
Comparison between the original ballast sample (left), high-resolution scan (center) and low-resolution (decimated) scan (right).

Blender, the open source animation, graphics, and modelling software suite, was primarily used to measure the sample's volume and surface area. The high-resolution STL file generated by the scanning process is imported into Blender followed by measurements using the 3^rd^ party *3D Print* add-on tool (enabled in the preferences menu). Once the model is selected in the software, the *Volume* and *Area* options of the 3D print tool are selected to calculate and display the volume and surface area of the ballast sample, respectively. Blender also provides a built-in decimation modifier which was used to reduce the density of the point cloud (and corresponding mesh), similar to the post-processing steps required during the scanning process. A decimation factor of 0.01 (1%) reduced the number of vertices and faces substantially, producing the final low-resolution version of the dataset. Fig. 12 illustrates a comparison between the original ballast sample (Fig. 12, left) and the difference in quality for the high- (Fig. 12, centre) and low-resolution models (Fig. 12, right).

## CRediT Author Statement

**André Broekman**: Conceptualization, Data Curation, Formal analysis, Investigation, Methodology, Project administration, Software, Validation, Visualization, Writing - Original Draft. **Jacobus Oostewald Van Niekerk:** Conceptualization, Investigation, Project administration, Resources, Visualization, Writing - Review & Editing. **Petrus Johannes Gräbe:** Funding acquisition, Resources, Supervision, Writing - Review & Editing.

## Declaration of Competing Interest

The authors declare that they have no known competing financial interests or personal relationships which have, or could be perceived to have, influenced the work reported in this article.
